# Potential Diagnostic and Prognostic Utility of miR-141, miR-181b1, and miR-23b in Breast Cancer

**DOI:** 10.3390/ijms21228589

**Published:** 2020-11-14

**Authors:** Mohamed Taha, Noha Mitwally, Ayman S. Soliman, Einas Yousef

**Affiliations:** 1 Department of Biochemistry, Faculty of Pharmacy, Cairo University, Cairo 11562, Egypt; nmtwally@dau.edu.sa; 2 Basic Medical Sciences Department, College of Medicine, Dar Al Uloom University, Riyadh 7222, Saudi Arabia; enasesawy@gmail.com; 3 Medical Physiology Department, Faculty of Medicine, Beni-Suef University, Beni Suef 62111, Egypt; Ayman.samaan@med.bsu.edu.eg; 4 Histology and Cell Biology Department, Faculty of Medicine, Menoufia University, Shebin Elkom 3251, Egypt

**Keywords:** miR-141, miR-181b1, miR-23b, breast cancer, bioinformatics

## Abstract

miRNAs, a group of short noncoding RNAs, are key regulators of fundamental cellular processes and signaling pathways. Dysregulation of miRNA expression with known oncogenic or tumor suppressor functions has been associated with neoplastic transformation. Numerous studies have reported dysregulation of miRNA-141, miR-181b1, and miR-23b in a wide range of malignancies, including breast cancer. To the best of our knowledge, no previous study had demonstrated the expression of miR-141-3p, miR-181b1-5p, and miR-23b-3p in different histological grades and molecular subtypes of breast cancer. Here, we identified differential expression of these three miRNAs in breast cancer tissues compared with benign breast fibroadenomas. In addition, high expression levels of miR-141-3p and miR-181b1-5p are strongly associated with aggressive breast carcinomas. We also confirmed the clinical potential of using the three miRNAs individually or combined as diagnostic and prognostic markers in breast cancer. Using bioinformatics analyses, we identified 23 hub genes of these three miRNAs which are involved in key signaling pathways in breast cancer. Furthermore, the KM plotter online database analysis demonstrates the association between elevated expression of miR-141 and miR-181b and shorter overall survival of breast cancer patients. Together, our data suggest an oncogenic role of the studied miRNAs and highlight their molecular roles and potential clinical applications in breast cancer.

## 1. Introduction

Breast cancers, which are heterogeneous and complex in nature, have been classified into different categories with unique genetic, epigenetic, and molecular characteristics [1]. Despite the recent advances in diagnosis and therapeutic approaches of breast cancer, some subsets remain deadly due to a high incidence of relapse and metastases. Benign breast diseases are non-cancerous disorders that encompass distinct histologic entities—non-proliferative, proliferative without atypia, and atypical hyperplasia. Although benign breast diseases have been recognizable as distinct from breast cancers, the association of these benign conditions to the development of breast cancer has been a controversial topic [2]. Therefore, identifying new biomarkers with diagnostic and prognostic values in breast cancer is urgently needed. Recently, many reports have demonstrated specific patterns of micro-RNA (miRNA) expression as potentially useful therapeutic targets, as well as diagnostic, and prognostic biomarkers in breast cancer [3,4,5].

miRNAs are a class of small, noncoding RNAs that vary in size from 19 to 25 nucleotides. They post-transcriptionally control gene expression by regulating mRNA through binding to complementary sequences with subsequent mRNA degradation and/or translational repression [6]. Dysregulation of miRNAs is often associated with cancer-related signaling pathways by functioning as oncogenes (oncomiRs) or tumor suppressor miRNAs [7,8,9]. To date, a growing number of studies have shown the association of aberrant miRNA expression with various types of cancer such as leukemia, prostate, lung, and breast cancer [10,11,12]. Among miRNAs that are implicated with breast tumorigenesis, miR-141, miR-23b, and miR-181b are critical regulators [13]. Functionally, overexpression of these three miRNAs significantly induces tumor growth, proliferation, and metastasis in breast cancer cell lines [14,15,16]. Despite many studies on mechanisms of miR-141, miR-181b, and miR-23b in breast cancer pathogenesis were carried out, their accurate expression in tissues of breast cancers has not yet been elucidated. To the best of our knowledge, no previous study has demonstrated the expression of miR-141, miR-23b, and miR-181b1 in different histological grades and molecular subtypes of breast cancer.

The aim of the current study was to assess the expression pattern and clinical significance of these three miRNAs in breast cancer tissues compared to benign breast fibroadenomas. Here, our results show that miR-141-3p, miR-181b1-5p, and miR-23b-3p are differentially expressed in breast cancer tissues compared with benign breast fibroadenomas. We demonstrated that high expression levels of miR-141-3p and miR-181b1-5p are strongly associated with highly aggressive breast carcinomas: grade III and triple-negative molecular subtype. Furthermore, we confirmed that using the three miRNAs individually or combined can serve as potential diagnostic and prognostic markers in breast cancer. Our data highlight the functional and clinical significance of these three miRNAs in breast cancer biology through bioinformatics analyses of their target genes and networks.

## 2. Results

### 2.1. miR-141-3p, miR-181b1-5p, and miR-23b-3p Are Differentially Expressed in Breast Cancer Tissues Compared with Benign Breast Fibroadenomas

**Figure 1:**
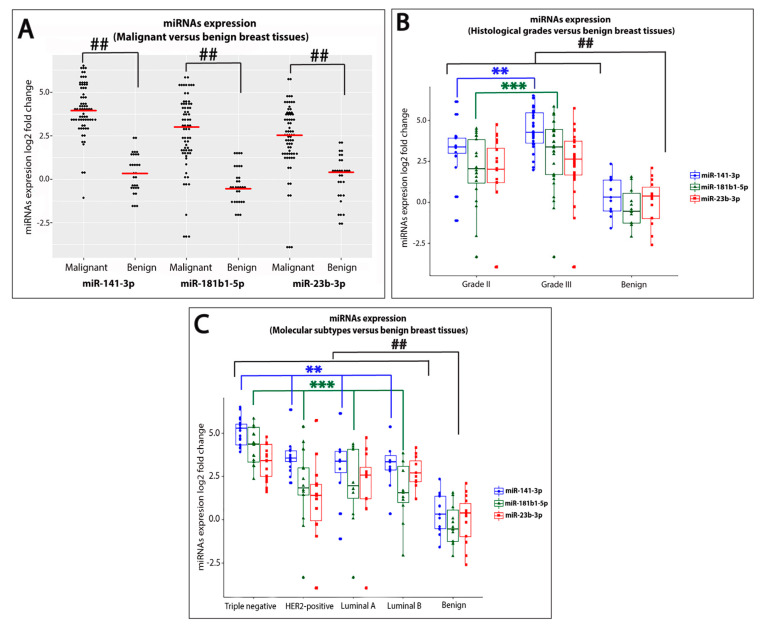
Comparisons of miR-141-3p, miR-181b1-5p, and miR-23b-3p in different groups of breast tissues. (**A**) Significant high expression levels (*p* < 0.0001) of miR-141-3p, miR-181b1-5p, and miR-23b-3p were detected in breast cancer tissues in comparison with benign breast fibroadenomas. (**B**) Significant high expression levels (*p* < 0.0001) of miR-141-3p, miR-181b1-5p, and miR-23b-3p were detected in grades II and III breast cancer when compared with benign breast fibroadenomas. Moreover, significant high expression (*p* < 0.05) of miR-141-3p and miR-181b1-5p was associated with high-grade breast tumors (grade III) in comparison to grade II breast cancer. (**C**) Significant high expression levels of miR-141-3p, miR-181b1-5p, and miR-23b-3p were detected in TNBC, HER2-positive, luminal A, and luminal B compared with benign controls. In addition, significant high expression (*p* < 0.05) of miR-141-3p and miR-181b1-5p in TNBC was detected compared with HER2-positive, luminal A, and luminal B subtypes. ^##^ indicates significant difference between malignant and benign breast tissues (*p* < 0.0001), ** indicates significant difference in miR-141-3p expression (*p* < 0.05), *** indicates significant difference in miR-181b1-5p expression (*p* < 0.05).

In the current study, we investigated the differential expression of miR-141-3p, miR-181b1-5p, and miR-23b-3p using qRT-PCR in specimens of 70 breast cancer patients in comparison to those of 30 benign breast fibroadenoma patients. The clinical features of the patients are listed in Table 1. Our results demonstrated significant upregulation (*p* < 0.0001) of the expression levels of the three miRNAs in breast cancer tissues compared with benign ones (fold change = 15.5, 7.95, 5.67, respectively) (Figure 1A). Spearman’s Rho correlation analysis was performed to evaluate the relationship between the three miRNAs expression in breast cancer tissues. As expected, significant (*p* < 0.0001) positive correlations were detected between the three miRNAs expression levels in breast cancer as follows: miR-141-3p and miR-181b1-5p: r = 0.59; miR-141-3p and miR-23b-3p: r = 0.60; and miR-23b-3p and miR-181b1-5p: r = 0.65.

To further explore the differential expression of these three miRNAs in breast cancer, we assessed their expression levels in breast cancer tissues of different histological grades in comparison to benign breast fibroadenomas. A significant upregulation of the three miRNAs in grades II and III breast cancer was detected when compared with benign breast tumors (Figure 1B). It is noteworthy that the levels of miR-141-3p, miR-181b1-5p, and miR-23b-3p expression were significantly upregulated (*p* < 0.0001) with 19.3, 10.2, and 6.17 folds, respectively, in grade III breast cancer compared with benign breast tissues.

Next, we studied the expression levels of these three miRNAs in different molecular subtypes of breast cancer compared to benign breast fibroadenomas. We defined the molecular subtypes using ER, PR, HER2, and Ki-67 as immunohistochemical surrogate markers as described by the St. Gallen’s Consensus [17]. Our results demonstrate significant upregulation of miR-141-3p and miR-181b1-5p in all molecular subtypes of breast cancer when compared with benign breast tumors (Figure 1C). For miR-23b-3p, although significant differences were detected between triple-negative breast cancer (TNBC), luminal A, and luminal B when compared with benign breast tumors, we did not find a significant difference with the HER2-positive molecular subtype. Furthermore, obvious high fold changes of miR-141-3p, miR-181b1-5p, and miR-23b-3p expression (39.1, 20.6, and 10.6 folds, respectively) were detected in TNBC in comparison with benign breast tissues.

### 2.2. Upregulation of miR-141-3p and miR-181b1-5p Expression Levels Is Strongly Associated with Highly Aggressive Breast Carcinomas

The high expression patterns of miR-141-3p, miR-181b1-5p, and miR-23b-3p in grade III and TNBC prompted us to pursue assessment of the three miRNAs in these two groups in comparison to other subtypes of breast cancer. The expression of miR-141-3p, miR-181b1-5p, and miR-23b-3p was then examined in breast cancer of various histological grades (grade II versus III). Significant high expression (*p* < 0.05) of miR-141-3p and miR-181b1-5p was associated with high-grade breast tumors (grade III) when compared with grade II breast cancer (Figure 1B). Of note, no significant difference was detected in the expression level of miR-23b-3p when comparing breast tumors from different histological grades.

We next assessed the expression of the three miRNAs in TNBC tissues in comparison to other breast cancer molecular subtypes. Significant high expression (*p* < 0.05) of miR-141-3p and miR-181b1-5p in TNBC was detected compared with HER2-positive, luminal A, and luminal B subtypes (Figure 1C). However, no significant difference could be identified in the expression level of miR-23b-3p when comparing TNBC with other molecular subtypes of breast cancer. These results suggested the prognostic ability of these miRNAs in discriminating grade III from grade II and TNBC from other molecular subtypes of breast cancer.

### 2.3. Roc Curve Analysis Revealed Diagnostic and Prognostic Utility of miR-141-3p, miR-181b1-5p, and miR-23b-3p in Breast Cancer

**Figure 2:**
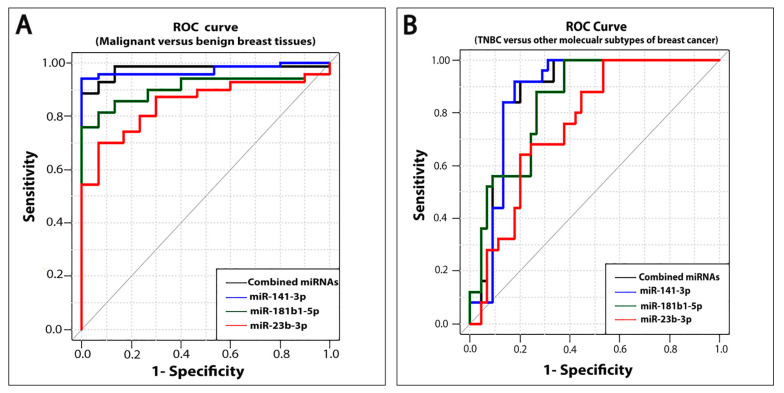
ROC curve analysis of miR-141-3p, miR-181b1-5p, and miR-23b-3p in different groups of breast tissues. (**A**) ROC curve analysis for miR-141-3p, miR-181b1-5p, and miR-23b-3p expression levels individually and combined to discriminate between patients with breast cancer and benign breast fibroadenomas; (**B**) ROC curve analysis for miR-141-3p, miR-181b1-5p, and miR-23b-3p individually and combined to distinguish TNBC from other molecular subtypes of breast cancer.

We used receiver operating characteristic (ROC) curve analysis to investigate the diagnostic power of miR-141-3p, miR-181b1-5p, and miR-23b-3p in breast cancer. Our analysis revealed the diagnostic utility of the three miRNAs, with area under the curve (AUC) of 0.97 (*p* < 0.0001) for miR-141-3p, 0.91 (*p* < 0.0001) for miR-181b1-5p, and 0.85 (*p* < 0.0001) for miR-23b-3p (Figure 2A). When we assessed the combination of miRNA-141-3p, miRNA-181b1-5p, and miRNA-23b-3p, our results showed that the AUC of the three miRNAs combined was 0.98 (*p* < 0.0001), which indicates excellent discriminatory power in separating breast cancer from benign tumors with optimal sensitivity and specificity (90% and 100%, respectively) (Table 2). Although any of the three miRNAs individually can discriminate malignant from benign breast tissues, our data suggest that the three miRNAs combined together have superior diagnostic ability, as they more effectively discriminate benign from neoplastic breast tumors.

Next, we used ROC curve analysis to investigate the prognostic power of miR-141-3p, miR-181b1-5p, and miR-23b-3p in discriminating TNBC from other breast cancer molecular subtypes. Our results demonstrate that these three miRNAs individually or combined can distinguish TNBC from other molecular subtypes of breast cancer with AUC of 0.88 (*p* < 0.0001) for miR-141-3p, 0.85 (*p* < 0.0001) for miR-181b1-5p, 0.76 (*p* < 0.0001) for miR-23b-3p, and 0.89 (*p* < 0.0001) for combined analysis (Figure 2B and Table 3). These results suggest that the combination of miR-141-3p, miR-181b1-5p, and miR-23b-3p exhibits superior prognostic power in distinguishing TNBC from other breast cancer molecular subtypes.

### 2.4. Prediction of miR-141-3p, miR-181b1-5p, and miR-23b-3p Target Genes in Breast Cancer

To predict the putative target genes of the three miRNAs, we used the miRDB online microRNAs target predication database [18]. All genes with a target score of ≥90 have been considered as differentially expressed genes (DEGs) [19]. Our analysis showed that 827 DEGs are target genes for miR-141-3p, miR-181b1-5p, and miR-23b-3p. In order to pinpoint genes associated with breast cancer from the identified DEGs, we employed the Pathway Studio^®^ web [20], an exhaustive resource of biological data to help better exploration of molecular interactions between molecules, cellular processes, and various diseases. Our analysis revealed that 310 target genes for these three miRNAs are linked to breast cancer (Supplementary Table S1).

### 2.5. Gene Ontology (GO) Functional Analysis and Pathway Enrichment Analysis for DEGs

To further understand the function and the biological mechanisms of the identified DEGs for miR-141-3p, miR-181b1-5p, and miR-23b-3p that are associated with breast cancer, we performed GO functional analysis and Kyoto Encyclopedia of Genes and Genomes (KEGG) pathway enrichment analysis using the freely accessible Database for Annotation, Visualization, and Integrated Discovery (DAVID) website [21,22]. The GO analysis classified the target genes of the studied miRNAs into three categories: biological process (BP), cellular component (CC), and molecular function (MF) [23]. Annotation results show that the identified DEGs are primarily enriched in the regulation of the phosphate metabolic process, regulation of phosphorylation, and regulation of cell motion. These biological processes are crucial for regulation of cancer cell metabolism, shape, and cell survival under stress. The five most significant (*p* < 0.05) enrichment terms of each GO category and the KEGG pathway enrichment analysis are listed in Table 4. Our findings from the KEGG pathway enrichment analyses reveal that miR-141-3p, miR-181b1-5p, and miR-23b-3p are mainly enriched in pathways in cancer, small-cell lung cancer, the ErbB-epidermal growth factor receptor (EGFR) signaling pathway, chronic myeloid leukemia, and mitogen-activated protein kinase (MAPK) signaling pathways. The complete GO analysis and KEGG pathway enrichment analysis for the identified DEGs are provided in Supplementary Table S2. These findings demonstrate that the DEGs, regulated by these three miRNAs, are involved in multiple biological processes and signaling pathways that may lead to invasiveness and metastasis of breast cancer cells.

### 2.6. Protein–Protein Interaction (PPI) Network Analysis, Identification of the Hub Genes, and Module Analysis

The identified 310 DEGs were then uploaded to the STRING online database to construct the PPI network [24]. Only PPI pairs with an association score of >0.4 were included in the created PPI network. The output data of the constructed PPI network (as .tsv) were afterwards imported to Cytoscape for further network analysis and identification of hub genes [25]. Our analysis using the Cytoscape plugin Molecular Complex Detection (MCODE) revealed 11 modules (clusters). Using the connectivity degree of ≥5 [26], we identified 23 hub genes in the top two significant modules: 10 in the first module and 13 in the second module (Supplementary Table S3).

**Figure 3:**
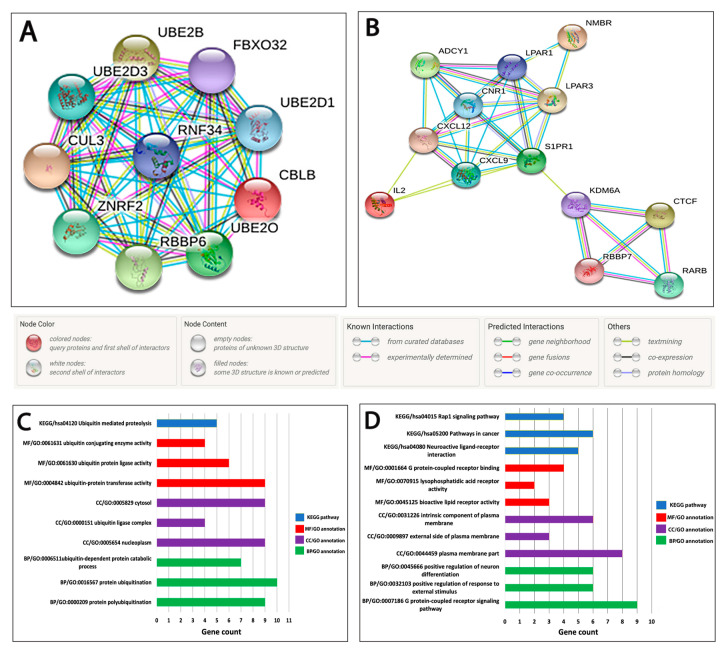
The PPI networks, GO annotations, and KEGG pathway enrichment analysis of identified hub genes of miR-141-3p, miR-181b1-5p, and miR-23b-3p. The PPI networks were created using the STRING online database for the identified hub genes obtained from the Cytoscape plugin MCODE analysis. (**A**) PPI network of the 10 hub genes in cluster 1; (**B**) PPI network of the 13 hub genes in cluster 2; *p* < 1.0 × 10^−16^ and *p* < 2.49 × 10^−14^ for the entire PPI network of modules 1 and 2, respectively; (**C**) bar plot illustrating the GO annotation and KEGG pathway enrichment analyses for cluster 1 hub genes; (**D**) bar plot illustrating the GO annotation and KEGG pathway enrichment analysis for cluster 2 hub genes. The blue, red, purple, and green bars represent the enrichment analysis results of KEGG, MF, CC, and BP, respectively.

To explore the PPI network of the identified hub genes, all genes in the two candidate modules were uploaded separately to the STRING database. Hub genes of module 1 form a single cluster of highly interconnected PPIs (Figure 3A), while genes of module 2 form two subclusters which are interconnected (Figure 3B). Each network node represents proteins produced by a single, protein-coding gene locus, while edges represent specific and meaningful protein-protein associations that contribute to a shared function. Furthermore, GO annotation and pathway enrichment analysis show the BP, CC, MF, and all the KEGG pathways in which the hub genes of each module were associated (Supplementary Table S3). The three most significant enrichment terms from each category of GO annotation and KEGG pathway enrichment analysis are shown in Figure 3C,D. This analysis revealed that the hub genes of module 1 are significantly involved in the regulation of protein ubiquitination such as ubiquitin–protein transferase activity, ubiquitin–protein ligase activity, and ubiquitin conjugating enzyme activity. Additionally, five genes of this module are highly enriched (FDR = 3.70 × 10^−8^) in the ubiquitin-mediated proteolysis pathway. On the other hand, hub genes in module 2 are highly involved in bioactive lipid receptor activity, lysophosphatidic acid receptor activity, and G-protein-coupled receptor binding. This module was also found to be significantly enriched with genes of the neuroactive ligand–receptor interaction pathway, pathways in cancer, and Ras-related protein 1 (Rap1) signaling pathways.

### 2.7. High Expression of miR-141 and miR-181b Is Associated with Worse Overall Survival (OS) in Breast Cancer Patients

**Figure 4:**
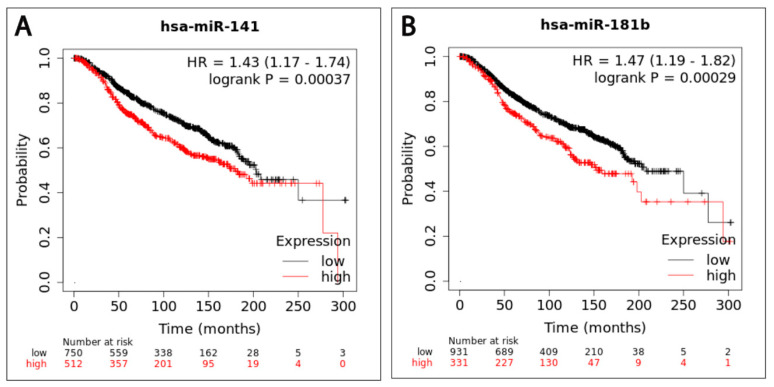
Overall survival curves for miR-141 and miR-181b in 1262 breast cancer patients using the KM plotter miRNA breast cancer online database. Kaplan–Meier curves are plotted for OS of breast cancer patients demonstrating that patients with high expression of (**A**) miR-141 and (**B**) miR-181b are associated with shorter overall survival.

We next examined the prognostic value and clinical significance of miR-141, miR-181b, and miR-23b expression in breast cancer using the Kaplan–Meier (KM) plotter miRNA breast cancer online database [27]. Among datasets included in the KM plotter, we employed the Molecular Taxonomy of Breast Cancer International Consortium (METABRIC) cohort dataset, which includes data from 1262 patients, to assess the association between the three studied miRNAs and OS in breast cancer patients [28]. In agreement with our results, for all 1262 patients, the OS is significantly lower in patients with high expression of both miR-141 (HR = 1.43, 95% CI = 1.17–1.74, *p* = 0.00037) and miR-181b (HR = 1.47, 95% CI = 1.19–1.82, *p* = 0.00029) compared with patients with low expressions (Figure 4A,B). No statistically significant difference was observed in the OS time between breast cancer patients with low and high expression of miR-23b (HR = 1.14, 95% CI = 0.94–1.39, *p* = 0.18). Hence, the KM plotter results emphasized the prognostic significance of miR-141 and miR-181b in breast cancer.

## 3. Discussion

Recent biomarker research advances have demonstrated the great potential for developing miRNAs as novel biomarkers and therapeutic targets in breast cancer. In the current study, we demonstrated differential expression of miR-141-3p, miR-181b1-5p, and miR-23b-3p in breast cancer tissues compared with benign breast tumors using qRT-PCR. Interestingly, high fold changes of miR-141-3p and miR-181b1-5p were detected in tumors with known aggressive behavior—grade III and triple-negative breast tumors—when compared with benign control. Furthermore, our results demonstrated the potential diagnostic and prognostic utility of these three miRNAs individually or combined in breast cancer. Lastly, we explored the biological functions and clinical significance of these three miRNAs in breast cancer using bioinformatics analyses.

Together with other previously reported data, our findings suggest the oncogenic role of the three miRNAs, as their high expression is detected in breast cancer tissues in comparison to benign breast fibroadenomas. Our results are consistent with both in vitro and in vivo studies that demonstrated the upregulation of miR-141, miR-181b1, and miR-23b expression in highly metastatic breast cancer cell lines [29,30,31,32]. Upregulation of miR-141 has been found to promote breast tumorigenesis through regulation of the phosphatidylinositol-4,5-bisphosphate 3-kinase/Protein kinase B (PI3K/AKT) signaling pathway, as well as the secretion of certain cytokines and growth factors [15]. Furthermore, it has been demonstrated that miR-181b overexpression has dual effects in inducing breast tumorigenesis and aggressiveness behavior, as it acts by (a) suppressing the expression of the proapoptotic Bim signal, causing dysregulation of the cell cycle, overgrowth, and the promotion of tumorigenesis [33,34], and (b) activating the most common signaling pathways of breast tumorigenesis, including IL6/STAT3 [35], transforming growth factor-β (TGF-β) [36], HIF-1 [34], and WNT/β-catenin [37]. Additionally, Jin et al. found that miR-23b is regulated by Her2/neu receptors and it can target tumor suppressor genes, leading to breast cancer initiation and progression [32].

Many previous studies are at odds with our results, as they demonstrated a tumor-suppressive role of miR-141, miR-181b, and miR-23 in different types of cancer such as lung, colon, prostate, and breast cancers [38,39,40,41]. For instance, other miRNA expression profiling studies reported downregulation of miR-141 and miR-23b in breast cancer tissues compared with matched surrounding tissues [39,42,43]. It was demonstrated by Wu et al. that miR-141 inhibits the growth and motility of hepatocellular carcinoma cells by targeting the Zinc finger E-box Binding homeobox 2 (ZEB2) gene [44]. Another study by Chen et al. reported the tumor suppressor role of miR-141 in renal cell carcinoma by inhibiting cell proliferation, migration, as well as invasion through targeting erythropoietin-producing hepatocellular A2 (EphA2) via modulating the EphA2/p-FAK/p-AKT/MMPs signaling cascade [45]. Similarly, miR-181b exerts its tumor suppressor effect in many cancers by promoting apoptosis and inhibiting cancer cell proliferation, migration, and invasion. These tumor suppressor effects of miR-181b are exerted by suppressing high-mobility group box-1 (HMGB1) and downregulation of NOVA alternative splicing regulator 1 [46,47]. Likewise, miR-23b was found to act as a tumor suppressor in bladder carcinoma by inhibiting tumor cell proliferation, colony formation, and migration and causing cell cycle arrest [48]. This discrepancy in miRNA expression in different studies can be explained by using different types of tissues and cell lines, different sample size, short- and long-term miRNA measurement together with employing different platforms of miRNA profiling techniques. Future studies with preclinical models will help solve this presumed contradiction.

To obtain further insights into the biological roles of miR-141-3p, miR-181b1-5p, and miR-23b-3p in breast cancer, bioinformatics and functional analyses were conducted using multiple bioinformatics platforms. Our KEGG enrichment analysis of the identified DEGs revealed that miR-141-3p, miR-181b1-5p, and miR-23b-3p regulated genes are involved in metabolic, biological, and cellular pathways in breast cancer. The most enriched pathways of these DEGs are cancer-related signaling pathways such as ErbB-epidermal growth factor receptor (EGFR) and mitogen-activated protein kinase (MAPK) signaling pathways. This is consistent with previous studies which demonstrated that miR-141 is an important modulator of the EGFR signaling pathway as it targets EGFR, preventing its translation in breast cancer cells [49,50]. Furthermore, Chiyomaru et al. reported that the miR-23b/27b cluster is involved in enhanced breast cancer cell proliferation, migration, and invasion through regulation of EGFR and c-Met signaling pathways [51]. In agreement with our KEGG pathway analysis, it was reported that dysregulation of MAPK leads to the loss of ER expression and plays a role in the development of the ER-negative breast cancer phenotype [52].

After a series of bioinformatic analyses, a total of 23 genes with a connectivity degree of ≥5 were considered as candidate hub genes that are regulated by miR-141-3p, miR-181b1-5p, and miR-23b-3p. The KEGG enrichment analysis of the identified 23 hub genes detected their involvement in critical cellular processes. The hub genes in module 1 are significantly involved in the ubiquitin-mediated proteolysis pathway, which has been previously proposed as a therapeutic target in different types of cancer, including breast cancer [53,54]. Interestingly, distinct breast cancer studies demonstrated that a number of fundamental proteins that act as oncogenes or tumor suppressor genes are significant participants in the ubiquitin–proteasome pathway [53,55]. Among the hub genes of the first module is Casitas B-lineage lymphoma-B (CBLB), a multifunctional adaptor protein and E3 ubiquitin ligase, which plays a role in regulating cell signaling. According to a previous in vitro study by Kang et al., the CBLB protein family promotes breast cancer development via inhibiting the tumor suppressor activity of TGF-β [56]. The hub genes in module 2 were significantly associated with neuroactive ligand–receptor interaction, pathways in cancer, and Ras-proximate-1 or Ras-related protein 1 (Rap1) signaling pathways. The neuroactive ligand–receptor interaction pathway involves many G-protein-coupled receptors and is one of the most common dysregulated pathways of malignancies, including breast cancer [57]. Rap1 is a small G protein that regulates diverse processes such as cell adhesion, cell–cell junction formation, and cell polarity, which are critical for cellular migration, invasion, and metastasis [58,59]. Moreover, the involvement of upregulated Rap1 in breast tumor formation and progression to malignancy has been reported in many studies [60,61]. Among the hub genes of module 2 is cannabinoid receptor-1 (CNR1), a component of neuroactive ligand–receptor interaction and Rap1 signaling pathways. Previous studies with human breast cancer cell lines demonstrated a positive correlation between CNR1 expression and the invasiveness of breast cancer [62]. The elevated expressions of Lysophosphatidic Acid Receptor 1 and 3 (LPAR1 and LPAR3), two other hub genes in module 2, are associated with poorly differentiated and advanced stages of breast cancer [63,64]. Taken together, these findings about the KEGG pathways and hub genes targeted by the three miRNAs provide further insight into the underlying biological processes and molecular mechanisms involved in initiation and progression of breast cancer. However, further analysis and validation of the molecular mechanisms are needed to confirm the clinical relevance of our findings.

Finally, the KM plotter was employed to evaluate the potential prognostic value of our studied miRNAs among 1262 patients with breast cancer. Our results confirmed that patients with elevated levels of miR-141 and miR-181b had shorter OS compared with patients with a lower expression of these miRNAs. These data strongly support the prognostic value of miR-141 and miR-181b in breast cancer. In alignment to our survival bioinformatics analysis, previous studies reported that high expression of miR-141 and miR-181 was markedly associated with shorter survival time of patients with breast cancer [65,66]. The worse OS associated with miR-141 and miR-181 can be explained by their suggested oncogenic role. Conversely, our results are completely at odds with Ping et al., who demonstrated that high expression of miR-141 was associated with better OS in TNBC patients [67]. One intrinsic limitation of our study is the small sample size, which did not allow us to study the associations between the three studied miRNAs and the clinical characteristics of the patients. In future research, it will be noteworthy to follow up on our patients’ survival, relapse, and metastasis to independently confirm the findings of our bioinformatics analysis. Much work remains to be done in order to further explore and better understand the role of these three miRNAs in human breast cancers and their clinical significance.

## 4. Materials and Methods

### 4.1. Tissue Samples and Ethical Approval

A total of seventy breast cancer tissue specimens and thirty mammary gland fibroadenomas were obtained from female patients undergoing a surgical procedure at Surgical Department of Kasr Alainy Teaching Hospital, Faculty of Medicine, Cairo University between March 2018 and September 2019. Written consent was obtained from all patients prior to tissue samples collection. Both malignant and benign tissues were sent for histopathological processing, diagnosis, and grading in the Pathology Department, Faculty of Medicine, Cairo University. Formalin-fixed paraffin-embedded (FFPE) blocks were prepared and stored in an environment with controlled humidity and temperature. Histological grades of breast cancer samples were evaluated using the modified Bloom and Richardson criteria [68]. ER, PR, HER2, and Ki-67 statuses were assessed using immunohistochemical staining and scored according to the American Society of Clinical Oncology/College of American Pathologists (ASCO/CAP) guidelines [69]. Molecular subtype classification was carried out as previously described [70,71]. Breast cancer patients with the following characteristics were excluded: (i) received preoperative neoadjuvant chemotherapy, immunotherapy, or radiation therapy; (ii) diagnosed with inflammatory breast cancer; (iii) had a prior history of cancer. All procedures were performed in accordance with the ethical guidelines of the Helsinki Declaration after approval from the research ethics committee at Cairo University, Cairo, Egypt (IRB# BC 2136).

### 4.2. RNA Isolation and Quantitative RT-PCR

For RNA extraction, 3–5 sections of 10 µm thick slices were prepared from each FFPE breast tissue sample. Total RNA was extracted from the FFPE breast tumor samples, after removing paraffin with xylene using a RNeasy Fibrous Tissue kit (Qiagen, cat. No. 74704, Valencia, CA, USA) as described in the manufacturer’s protocol. The purity and concentration of RNA were evaluated using a NanoDrop ND-100 Spectrophotometer (Thermo Scientific, Waltham, MA, USA). Then, we converted total RNA to complementary DNA (cDNA) using the miScript II RT kit (Qiagen, cat. No. 218161, Valencia, CA, USA) as described in the manufacturer’s protocol. qRT-PCR was performed using the SYBR Green PCR Super Mix (Qiagen, cat. No. 218300, Valencia, CA, USA) and the specific microRNA Locked Nucleic Acid PCR primers (Qiagen, cat. No. 218300, Valencia, CA, USA). Specific miScript forward primers were as follows: mir-141-3p: 5′-UAACACUGUCUGGUAAAGA UGG-3′, mir-181b1-5p: 5′-AACAUUCAUUGCUGU CGGUGGG U-3′, miR-23b-3p: 5′-AUCACAUUGCCAGG GAUUACC-3′; the reverse primers were universal. We used the BIO-RAD CFX96 Real-Time PCR Detection System (Bio-Rad, Hercules, CA, USA) to perform qRT-PCR. The PCR parameters were as follows: 95 °C for 15 min, followed by 40 cycles of 94 °C for 15 s, 55 °C for 30 s, and 70 °C for 30 s. Each sample was examined in triplicate and the expression of miRNAs was defined based on the threshold cycle (Ct); relative expression levels were normalized using the ΔΔC_t_ equation with respect to RNU6B internal control [72].

### 4.3. Bioinformatics

#### 4.3.1. Target Genes Prediction in Breast Cancer

To predict target genes of miR-141-3p, miR-181b1-5p, and miR-23b-3p, we used the miRDB (http://mirdb.org/) online microRNA target prediction database on 7 August 2020 [18]. We selected the genes with a target score of ≥90 for further filtration and network analysis [19]. In order to identify DEGs involved in breast cancer, the gene list with a target score of ≥90 generated from miRDB was imported to the Pathway Studio Web (Elsevier, Netherlands) (http://www.pathwaystudio.com) [20]. This software is an exhaustive resource of biological data extracted from published scientific research to help better exploration of molecular interactions between molecules, cellular processes, and various diseases. In our study, pathway studio analyses were conducted to find molecular interactions between the DEGs detected from miRDB and breast cancer.

#### 4.3.2. GO Functional Analysis and Pathway Enrichment Analysis of Breast Cancer DEGs

To understand the potential biological functions and molecular mechanisms of the detected DEGs, we performed a GO functional analysis and a KEGG pathway enrichment analysis using the freely accessible DAVID database (http://david.ncifcrf.gov/) (version 6.8) [21,22]. GO functional analysis included assessing the BP, CC, and MF that might be affected by miR-141-3p, miR-181b-5p, and miR-23b-3p [23]. In addition, enrichment analysis was used to identify KEGG pathways associated with identified DEGs in breast cancer. Statistically significant difference was considered for *p* < 0.05.

#### 4.3.3. PPI Network Construction, Identification of the Hub Genes, and Module Analysis

We submitted the retrieved DEGs that are targets of miR-141-3p, miR-181b1-5p, and miR-23b-3p to the STRING database (version 11.0) (https://string-db.org/) on 9 August 2020 to construct the PPI network [24]. STRING is a database that allows retrieval of known and predicted protein-protein interactions from a variety of sources such as genomic context predictions, high-throughput lab experiments, co-expression, automated text mining, and previous knowledge in databases [24]. The aim of PPI network is to summarize the predicted association for a group of proteins, where the nodes represent the protein and the edges represent the predicted functional associations. We used a medium confidence score of >0.4 as a threshold to construct the PPI network, where only interactions above this score were included in the constructed network. The output data for the PPI interaction network were next exported as tab separated values (.tsv format) and then imported to Cytoscape (version 3.8.0), an open source software platform, for further network analysis [25]. In Cytoscape, we applied the clustering algorithm MCODE to explore the gene–gene connectivity through focusing in highly interconnected nodes that often possess specific biological functions known as clusters or modules. The criteria were set as follows: MCODE score cutoff = 0.2, k-core = 2, max. depth from seed = 100, and degree cutoff = 2. We considered genes with a connectivity degree (MCODE score) of ≥5 as hub genes [26]. Subsequently, GO functional analysis and KEGG pathway enrichment analysis of the identified hub genes were carried out using the STRING database; *p* < *0*.05 was considered to be significantly different.

#### 4.3.4. OS Analysis of miR-141, miR-181b, and miR-23b in Breast Cancer Patients

We further used the KM plotter [27], an integrated online bioinformatics tool, to assess the prognostic significance of miR-141, miR-181b, and miR-23b in patients with breast cancer. In the current analysis, we used the METABRIC cohort online dataset via the KM plotter miRNA breast cancer online database, which is characterized as follows: it includes data from 1262 patients, long follow-up (median: 94 months), average characteristics (78% ER positive, 12% HER2-positive), and available treatment data [28]. To analyze the prognostic value of the three miRNAs, the patient samples were split into two groups according to the auto select best cutoff feature of each proposed miRNA, where the median survival was reported in months. The two patient cohorts were compared by a Kaplan–Meier survival plot, and the hazard ratio with 95% confidence intervals and log rank *p*-value were calculated. A *p*-value of <0.05 was considered statistically significant. The entire analysis of the KM plotter was performed using R 2.15.0 statistical software.

### 4.4. Statistical Analysis

At least three independent measurements of miRNAs were conducted. Our data analysis was performed using R version 3.6.1. All tests were done after the normality was tested with the Shapiro–Wilk normality test. Levels of miRNA expression in histological grades and molecular subtypes of breast cancer were described as median fold change in comparison with benign cases by the Kruskal–Wallis rank sum test and the Dunn (1 964) Kruskal–Wallis multiple comparison test. Levels of miR-141-3p, miR-181b1-5p, and miR-23b-3p expression were measured using qRT-PCR in the breast tissue specimens and normalized to RNU6B mRNA as the reference gene. The fold change was calculated as the ratio of miRNA expression to the internal control. Of note, log2 values were used to visualize the gene expression data for easier data interpretation. The correlations between the studied miRNA expressions were evaluated using Spearman’s Rho correlation coefficient. To assess the diagnostic and prognostic accuracy of miR-141-3p, miR-181b1-5p, and miR-23b-3p, ROC curve analysis was conducted and the AUC was calculated [73]. *p* < 0.05 was considered to indicate a statistically significant difference.

## 5. Conclusions

To sum up, our results clearly emphasize the diagnostic and prognostic roles of miR-141-3p, miR-181b1-5p, and miR-23b-3p individually or combined in breast cancer. Upregulation of the three miRNA expressions was detected in breast cancer tissues compared with benign breast fibroadenomas. High expression levels of miR-141-3p and miR-181b1-5p are strongly associated with highly aggressive breast carcinomas. Furthermore, KM plotter online database analysis confirmed the association of elevated levels of miR-141 and miR-181b with shorter survival time of breast cancer patients. By using different tools of bioinformatics and target prediction analyses, we identified many potential miRNA regulatory pathways, which may improve our understanding of the molecular and biological mechanisms of breast cancer. Further work is needed using a larger cohort of patients in order to validate the potential clinical uses of these three miRNAs as biomarkers in breast cancer.

## Figures and Tables

**Table 1:** Clinicopathological characteristics of patients included in the study.

Clinicopathological Characteristics	N (%)
Benign fibroadenomas (control)	30 (30%)
Infiltrating ductal carcinomas	70 (70%)
Age	
29–40	12 (17.14%)
41–60	28 (40%)
+60	30 (42.86%)
Histological subtypes	
Ductal	49 (70%)
Lobular	21 (30%)
Histological Grade	
I	0
II	24 (34.3%)
III	46 (65.7%)
Estrogen receptor status	
Positive	47 (67.14%)
Negative	23 (32.86%)
Progesterone receptor status	
Positive	23 (32.85%)
Negative	47 (67.15%)
HER2 status	
Positive	26 (37.14%)
Negative	44 (62.86%)
Ki-67	
<14%	12 (17.14%)
≥14%	58 (82.86%)
Molecular Subtypes	
Luminal A	12 (17.14%)
Luminal B	11 (15.71%)
HER2-positive	22 (31.43%)
Triple negative	25 (35.72%)

**Table 2:** ROC analysis of miRNAs in patients with breast cancer compared with benign controls.

miRNA	Sensitivity	Specificity	Cutoff	AUC	*p*-Value
miR-141-3p	0.94	1	4.3	0.97	<0.0001
miR-181b1-5p	0.76	1	2.98	0.91	<0.0001
miR-23b-3p	0.7	0.93	2.99	0.85	<0.0001
Combined	0.90	1	12.17	0.98	<0.0001

**Table 3:** ROC analysis of miRNAs in TNBC patients compared with other molecular subtypes of breast cancer.

miRNA	Sensitivity	Specificity	Cutoff	AUC	*p*-Value
miR-141-3p	0.92	0.82	17.02	0.88	<0.0001
miR-181b1-5p	1	0.62	5.1	0.85	<0.0001
miR-23b-3p	1	0.47	2.91	0.76	0.0001
Combined	0.92	0.80	43.39	0.89	<0.0001

**Table 4:** GO functional and pathway enrichment analyses of DEGs in breast cancer.

Category	Term	Number of Enriched Genes	%
BP_FAT	GO:0019220 regulation of phosphate metabolic process	36	12.7
BP_FAT	GO:0051174 regulation of phosphorus metabolic process	36	12.7
BP_FAT	GO:0042425 regulation of phosphorylation	35	12.3
BP_FAT	GO:0006357 regulation of transcription from RNA polymerase II promoter	42	14.8
BP_FAT	GO:0051270 regulation of cell motion	21	7.4
CC_FAT	GO:0005654 organelle lumen	47	12
CC_FAT	GO:0031981 nuclear lumen	44	15.5
CC_FAT	GO:0044459 plasma membrane part	58	20.4
CC_FAT	GO:0044451 nucleoplasm part	21	7.4
CC_FAT	GO:0070013 intracellular organelle lumen	46	16.2
MF_FAT	GO:0030528 transcription regulator activity	62	21.8
MF_FAT	GO:0003700 transcription factor activity	46	16.2
MF_FAT	GO:0004672 protein kinase activity	30	10.6
MF_FAT	GO:0004674 protein serine/threonine kinase activity	23	8.1
MF_FAT	GO:0016564 transcription repressor activity	19	6.7
KEGG_PATHWAY	hsa05200: Pathways in cancer	24	8.5
KEGG_PATHWAY	hsa05222: Small cell lung cancer	11	3.9
KEGG_PATHWAY	hsa04012: ErbB signaling pathway	11	3.9
KEGG_PATHWAY	hsa05220: Chronic myeloid leukemia	10	3.5
KEGG_PATHWAY	hsa04010: MAPK signaling pathway	17	6

BP, biological process; CC, cellular component; MF, molecular function; FAT, functional annotation table; KEGG, Kyoto Encyclopedia of Genes and Genomes; GO, Gene ontology; ErbB, epidermal growth factor; MAPK, mitogen-activated protein kinases, (%) indicates the percentage of involved genes/total genes.

## Supplementary Materials

Supplementary materials can be found at https://www.mdpi.com/1422-0067/21/22/8589/s1.

## Abbreviations

ASCO/CAP	American Society of Clinical Oncology/College of American Pathologists
AUC	Area Under the Curve
BP	Biological Process
CC	Cellular Component
CBLB	Casitas B-Lineage Lymphoma-B
CNR1	Cannabinoid Receptor-1
DEGs	Differentially Expressed Genes
DAVID	Database for Annotation, Visualization, and Integrated Discovery
EGFR	Epidermal Growth Factor Receptor
EphA2	Erythropoietin-Producing Hepatocellular A2
FFPE	Formalin-Fixed Paraffin-Embedded
GO	Gene Ontology
HMGB1	High-Mobility Group Box-1
KEGG	Kyoto Encyclopedia of Genes And Genomes
KM	Kaplan–Meier
MF	Molecular Function
MAPK	Mitogen-Activated Protein Kinase
MCODE	Molecular Complex Detection
METABRIC	Molecular Taxonomy of Breast Cancer International Consortium
OS	Overall Survival
PPI	Protein–Protein Interaction
PI3K/AKT	Phosphatidylinositol-4,5-Bisphosphate 3-Kinase/Protein Kinase B
ROC	Receiver Operating Characteristic
Rap1	Ras-Related Protein 1
TNBC	Triple-Negative Breast Cancer
TGF-β	Transforming Growth Factor-Β
ZEB2	Zinc Finger E-Box Binding Homeobox 2
